# Emergence of decapod hepanhamaparvovirus genotype V and its co-infection with *Enterocytozoon hepatopenaei* in cultured *Penaeus vannamei* in Thailand: Evidence from epidemiological, pathogenicity, and microbiome analyses

**DOI:** 10.14202/vetworld.2025.3496-3508

**Published:** 2025-11-23

**Authors:** Onanong Charoenwai, Pornpawit Tanpichai, Pimwarang Sukkarun, Hye Jin Jeon, Bumkeun Kim, Jee Eun Han, Patharapol Piamsomboon

**Affiliations:** 1 Graduate Program in Veterinary Science and Technology International Program, Faculty of Veterinary Science, Chulalongkorn University, Bangkok, 10330, Thailand; 2 Aquatic Animals Clinic, Faculty of Veterinary Medicine, Mahanakorn University of Technology, Bangkok, 10530, Thailand; 3 Faculty of Veterinary Science, Rajamangala University of Technology Srivijaya, Nakhonsithammarat, 80240, Thailand; 4 Institute for Veterinary Biomedical Science, College of Veterinary Medicine, Kyungpook National University, Daegu, 41566, Republic of Korea; 5 Department of Veterinary Medicine, Faculty of Veterinary Science, Chulalongkorn University, Bangkok, 10330, Thailand

**Keywords:** decapod hepanhamaparvovirus genotype V, *Enterocytozoon hepatopenaei*, microbiome, pathobiome, *Penaeus vannamei*, Thailand aquaculture

## Abstract

**Background and Aim:**

Growth retardation syndrome in cultured *Penaeus vannamei* has been associated with *Enterocytozoon hepatopenaei* (EHP) and a recently identified decapod hepanhamaparvovirus (DHPV) genotype V. However, data on its prevalence, pathogenicity, and interaction with the shrimp hepatopancreatic microbiome in Thailand remain limited. This study aimed to determine the incidence and co-infection rate of DHPV genotype V with EHP, evaluate its pathogenic potential, and explore microbiome alterations associated with infection.

**Materials and Methods:**

Between 2022 and 2023, 1,270 shrimp from 127 grow-out ponds across 46 farms in eastern Thailand and post-larvae 12 from five hatcheries in the south were screened for DHPV and EHP by polymerase chain reaction. Six representative isolates underwent phylogenetic analysis based on non-structural protein 1 (*NS1*) and *NS2* genes. Pathogenicity was evaluated by immersion challenge bioassays in specific pathogen-free *P. vannamei*. Hepatopancreatic microbiomes of naturally infected and healthy shrimp were compared using *16S ribosomal RNA* gene sequencing and Quantitative Insights Into Microbial Ecology 2-based analysis.

**Results:**

DHPV was detected in 54.33% (69/127) of ponds and 4% (1/25) of hatchery tanks. Co-infection with EHP occurred in 40.16% of ponds. Phylogenetic analysis showed 97.99%–98.82% similarity with DHPV genotype V from South Korea, confirming transboundary genetic relatedness. Experimental infection caused low mortality (20%) but resulted in viral replication (10^1^–10^3^ copies/μL) and characteristic intranuclear inclusion bodies in hepatopancreatic cells. DHPV-infected shrimp exhibited distinct microbiome profiles with elevated Firmicutes, Planctomycetota, and Actinobacteriota abundances, supporting a pathobiome shift during infection.

**Conclusion:**

This is the first report of DHPV genotype V in *P. vannamei* from Thailand and its frequent co-infection with EHP. Despite its low experimental virulence, the widespread occurrence and microbiome dysbiosis suggest that it may have subclinical impacts that could exacerbate growth retardation. Routine molecular screening in hatcheries and farms, coupled with integrated viral–microbiome surveillance, is essential for sustainable aquaculture biosecurity and aligns with the United Nations Sustainable Development Goal 14 (Life Below Water) by promoting resilient aquatic food systems.

## INTRODUCTION

Shrimp aquaculture in Thailand has faced major challenges due to several well-documented diseases, including white spot syndrome virus (WSSV) [1], yellowhead virus [2], decapod iridescent virus I [3], acute hepatopancreatic necrosis disease (AHPND) [4], and *Enterocytozoon hepatopenaei* (EHP) infection [5]. Among these, viral pathogens remain the most devastating, posing significant threats to global shrimp production and farm profitability [6]. Hepatopancreatic parvoviruses (HPV), historically referred to as “historical HPV” [7], are known to infect the epithelial cells of the hepatopancreas and midgut of penaeid shrimp. Infected shrimp typically exhibit nonspecific gross clinical signs such as hepatopancreatic atrophy, anorexia, retarded growth, and reduced grooming behavior. Although HPV infections have long been associated with production losses, the virus is rarely detected as a single causative agent. Instead, it is frequently found in combination with opportunistic pathogens, forming part of multi-agent epizootics in cultured shrimp populations [8–10].

Recently, a novel decapod hepanhamaparvovirus (DHPV) genotype V has emerged as a potential threat to shrimp health, particularly in the Pacific white shrimp *Penaeus vannamei*, with confirmed reports from India and South Korea [11, 12]. Taxonomically, DHPV has been reclassified as a single species, DHPV 1, within the family *Parvoviridae* and subfamily *Hamaparvovirinae* [13, 14]. It is a small, non-enveloped, icosahedral virus possessing a linear, negative-sense single-stranded DNA genome ranging from 4 kilobase pair (kbp) to 6 kbp [15]. Co-infections involving DHPV and other pathogens have become increasingly recognized as aggravating factors in shrimp health management. Shrimp are highly susceptible to simultaneous or sequential infections, which can cause greater economic losses than single-agent infections [16]. DHPV has been detected in co-infections with monodon baculovirus in *Penaeus monodon* [17], as well as with WSSV and EHP in *P. vannamei* [12, 18]. EHP, an intracellular microsporidian parasite, infects hepatopancreatic and midgut epithelial cells, resulting in reduced feed intake and severe growth retardation [5, 19]. Notably, a high incidence of DHPV infection has been reported among shrimp exhibiting growth retardation, both in the presence and absence of EHP infection, indicating that the role of DHPV in this syndrome may have been underestimated [12]. Despite the increasing recognition of DHPV as an emerging pathogen in shrimp aquaculture, information on its epidemiology, genotype distribution, and co-infection dynamics within cultured *P. vannamei* populations in Thailand remains scarce. Most prior studies have focused on DHPV occurrences in *P. monodon* or have originated from other Asian regions such as India and South Korea [11, 12], leaving a critical knowledge gap regarding its geographical distribution, genetic diversity, and potential impact under intensive Thai aquaculture systems. Furthermore, the interactive effects of DHPV with EHP, a major cause of growth retardation, have not been fully elucidated. Given that both pathogens target hepatopancreatic tissues, their co-infection could synergistically alter host physiology and microbial homeostasis, yet comprehensive pathobiome-based studies remain limited. Similarly, the influence of DHPV on the hepatopancreatic microbiome and its implications for shrimp health and resilience are poorly understood. These gaps hinder the development of effective diagnostic, preventive, and biosecurity measures for sustainable shrimp farming in Thailand.

This study aimed to investigate the incidence, co-infection rate, and genetic characteristics of DHPV genotype V in cultured *P. vannamei* populations in Thailand, with a particular focus on its association with EHP. In addition, the study sought to evaluate the pathogenic potential of DHPV genotype V through experimental immersion challenges and to characterize the hepatopancreatic microbiome of naturally infected shrimp to understand microbial dysbiosis and pathobiome alterations. By integrating epidemiological, molecular, and microbiome analyses, this research provides a holistic understanding of DHPV–EHP co-infections and their implications for shrimp health and aquaculture productivity. The findings contribute to Sustainable Development Goal (SDG) 14 (Life Below Water) by promoting responsible aquaculture practices that safeguard aquatic biodiversity and ecosystem health, and to SDG 2 (Zero Hunger) by supporting disease-resilient food production systems. Establishing baseline data on DHPV genotype V distribution and its pathobiological interactions will strengthen Thailand’s aquatic animal health surveillance and guide future strategies for sustainable shrimp farming and biosecurity management.

## MATERIALS AND METHODS

### Ethical approval and informed consent

All experimental procedures involving shrimp, including study design to minimize animal distress and sample numbers, euthanasia, biosafety, waste management, and water-disposal protocols, were conducted in accordance with the ethical standards of the Chulalongkorn University Animal Care and Use Committee (CU-ACUC Approval No. 2531101). The study adhered to the principles and guidelines outlined in the World Organisation for Animal Health (WOAH, 2024) Manual of Diagnostic Tests for Aquatic Animals for the handling and diagnostic testing of aquatic species. Sampling from grow-out ponds and hatcheries was conducted under the supervision of a licensed veterinarian from the Department of Veterinary Medicine, Faculty of Veterinary Science, Chulalongkorn University, Thailand. Shrimp were obtained with prior informed consent from farm owners, and all experimental bioassays using specific-pathogen-free *P. vannamei* were performed in biosecure facility at the Faculty of Veterinary Science, Chulalongkorn University.

### Study period and location

The study was conducted from September 2022 to May 2023 at the Department of Veterinary Medicine, Faculty of Veterinary Science, Chulalongkorn University, Thailand.

### Sample collection and screening

#### Shrimp sampling

A total of 127 ponds with a history of growth retardation were sampled from 46 intensive *P. vannamei* grow-out farms located in eastern Thailand. Ten shrimp were collected from each pond, with each farm consisting of one to eight ponds.

Additionally, samples from five hatcheries in southern Thailand were included for routine disease monitoring. Thirty post-larvae (PL12) were collected from each tank (stock density: 100,000 PL/m³) and pooled as one sample, resulting in a total of 25 tanks (five tanks per hatchery). Sampling procedures followed the guidelines of the Department of Fisheries, Ministry of Agriculture and Cooperatives, Thailand.

#### Microscopic examination for EHP

For EHP screening, five shrimp per pond were examined by preparing fresh hepatopancreas mounts and observing them under a light microscope (Olympus CX23; Olympus Optical Co. Ltd., Japan) with 1,000× magnification. All microscopic examinations were performed by trained aquaculture professionals from the Department of Fisheries, Thailand.

#### Sample transport and storage

All shrimp samples were transported on ice at 4°C to the Faculty of Veterinary Science, Chulalongkorn University, within 4 h of collection and stored at −80°C until further analysis.

### DNA extraction and polymerase chain reaction (PCR)

#### DNA extraction

Ten shrimp per pond were pooled as one sample, and approximately 25 mg of hepatopancreas tissue was collected for EHP and DHPV assays. For shrimp PL12, carapaces were pooled until 25 mg of tissue was obtained. DNA was extracted using a Nucleospin Tissue Mini Kit (Catalog No. DMCN-740952.50, Macherey-Nagel, Düren, Germany) according to the manufacturer’s instructions.

DNA concentration and purity were determined using a NanoDrop One/OneC Microvolume UV-Vis spectrophotometer (Catalog No. ND-ONE-W, Thermo Scientific, USA). Samples with concentrations ≥50 ng/μL and 260/280 absorbance ratios between 1.8 and 2.0 were stored at −80°C before analysis.

#### Detection of EHP

EHP detection was conducted using nested PCR targeting the spore wall protein (*SWP*) gene [20]. The first-round reaction (25 μL total volume) contained 0.2 μM of each primer, 12.5 μL of 2X DreamTaq PCR Master Mix (Catalog No. K1071, Thermo Fisher Scientific), 1 μL of DNA template (50–100 ng), and nuclease-free water.

Distilled water and genomic DNA extracted from EHP-infected tissue served as negative and positive controls, respectively. The housekeeping gene elongation factor 1α (*EF-1α*) was used as an internal control [21].

#### Detection of DHPV

DHPV was detected using PCR assays targeting the non-structural protein 2 (*NS2*) and *NS1* genes [22], under the same reaction conditions described above. The primers and PCR cycling conditions are listed in Table S1.

DHPV-positive amplicons were sequenced for genotypic identification using Sanger sequencing with BigDye chemistry, achieving a quality score of 99 (Macrogen Inc., Seoul, South Korea).

### Phylogenetic analysis of DHPV

Nucleotide sequences obtained from six representative DHPV-positive samples were compared with homologous sequences using the BLAST tool (National Center for Biotechnology Information; https://blast.ncbi.nlm.nih.gov/Blast.cgi).

Sequences were deposited in GenBank under accession numbers: B452 (PQ222688), B454, B468, B511, B577-2, and B587 (PX445146–PX445150). Sequence alignment was performed using CLUSTALW, and a phylogenetic tree was constructed in MEGA version 12 (Pennsylvania State University, USA) (https://www.megasoftware.net.) [23] employing the neighbor-joining method with the Tamura-Nei model and 1,000 bootstrap replicates. The resulting tree was visualized using the Interactive Tree of Life (iTOL, version 6) (European Molecular Biology Laboratory (EMBL), Germany) (https://itol.embl.de) [24].

### Pathogenicity of DHPV genotype V

#### Preparation of viral suspension

Field-collected DHPV-infected shrimp were used for immersion challenge bioassays. Viral copy numbers in infected tissues were quantified by real-time PCR (quantitative PCR [qPCR]). Primers and a TaqMan probe were designed using Primer Express 3.0.1 based on a partial DHPV sequence from *P. vannamei* (Accession No. ON015651) [25].

Each qPCR reaction (20 μL) contained 0.5 μM of each primer, 0.25 μM of TaqMan probe, 10 μL of 2X AccuPower Plus DualStar qPCR Master Mix (Catalog No. K-6603, Daejeon, Republic of Korea), and 1 μL of 100 ng DNA template. Cycle threshold (Ct) values were used to calculate viral copy numbers.

Hepatopancreatic tissues containing viral loads >10^7^ copies/μL were used to prepare viral suspensions. Approximately 1 g of infected hepatopancreas was homogenized with sterile sand in 1X phosphate-buffered saline (PBS) to a total volume of 10 mL. The homogenate was centrifuged at 750 × *g* for 10 min, and the supernatant was stored at −80°C. Viral load in the suspension was verified by qPCR before use in bioassays.

#### Immersion challenge bioassay

Eighty specific-pathogen-free *P. vannamei* (0.4 ± 0.3 g) were obtained from a Good Agricultural Practice-certified hatchery and confirmed negative for EHP. A double-blind experimental design was used.

Shrimp were randomly distributed into eight 5-L tanks (10 shrimp per tank), forming two groups: treatment and control (four tanks each). Treatment shrimps were immersed in 10 mL of DHPV suspension per 1 L of water (final viral load: 10^5^ copies/μL) for 24 h, while control shrimp were exposed to 1X PBS. After exposure, each shrimp was transferred to a separate 1 L aquarium to prevent cannibalism.

Tanks were maintained with aerated artificial seawater at 18 ppt salinity and 28°C–30°C, with 50% water exchange and daily waste removal. Shrimp were fed a commercial diet (3% body weight/day, divided into 3 feedings). Mortality and behavior were monitored for 14 days.

At the end of the trial, shrimp were euthanized using 100 parts per million clove oil (Aquanes, Better Pharma, Thailand) for 10 min. Hepatopancreatic tissues were collected for DHPV detection by PCR and qPCR, fixed in AFA solution for 24 h, embedded in paraffin, sectioned at 5 μm, and stained with hematoxylin and eosin following standard histological methods [7, 26].

### Analysis of the hepatopancreatic microbiome

#### DNA extraction from hepatopancreas

The hepatopancreatic microbiomes of DHPV-positive and DHPV-negative shrimp were analyzed from a single farm under uniform management conditions (feeding and water quality). Four shrimp per pond were screened to minimize biological variation.

All selected shrimp were free from EHP and other known shrimp pathogens listed in the *Manual of Diagnostic Tests for Aquatic Animals* [27]. Genomic DNA was extracted from individual samples and assessed for quality and quantity before *16S ribosomal RNA* sequencing. Distilled water served as a negative extraction control.

#### 16S rRNA gene sequencing

Library preparation and sequencing were performed by Macrogen Inc. (San Diego, USA) using the Illumina MiSeq platform to generate 301 base pair paired-end reads. The V3–V4 regions of the bacterial *16S rRNA* gene were amplified using primers 341F (TCGTCGGCAGCGTCAGATGTGTATAAGAGACAGCCTACGGGNGGCWGCAG) and 805R (GTCTCGTGGGCTCGGAGATGTGTATAAGAGACAGGACTACHVGGGTATCTAATCC).

The resulting sequences were deposited in the National Center for Biotechnology Information Sequence Read Archive (BioProject PRJNA1180691; https://www.ncbi.nlm.nih.gov/sra/PRJNA1180691).

#### Bioinformatic and statistical analyses of the microbiome

Raw reads were quality-checked using FastQC (v0.12.1) (Babraham Bioinformatics, United Kingdom) (https://www.bioinformatics.babraham.ac.uk/projects/fastqc/) [28] and MultiQC (v1.17) (National Genomics Infrastructure, Sweden) (http://multiqc.info) [29]. Primer sequences were trimmed with Cutadapt (v4.5) (National Genomics Infrastructure) (https://cutadapt.readthedocs.io/en/stable/index.html) [30], and reads with Phred scores <28 were removed. Data preprocessing, taxonomic assignment, and diversity analyses were conducted in Quantitative Insights Into Microbial Ecology (QIIME2) (v2023.7) (Northern Arizona University, USA) (https://library.qiime2.org) [31].

Paired-end reads were merged, chimeras removed through Divisive Amplicon Denoising Algorithm 2 (DADA2), an open-source R package (https://github.com/benjjneb/dada2) [32], and mitochondrial/chloroplast sequences excluded. Taxonomic identification was performed using the SILVA database (v138) (Leibniz Institute DSMZ, Germany) (https://www.arb-silva.de) [33]. Sequence abundance was normalized through rarefaction based on the smallest sample depth [34].

Alpha diversity was evaluated using the Simpson index, and beta diversity (Jaccard similarity) was analyzed to assess inter-group differences. Principal coordinate analysis (PCoA) was performed to visualize community separation.

Visualization was performed using R (v4.3.2) packages ggplot2 (The R Core Team, Austria) (https://CRAN.R-project.org/package=ggplot2) [35] and pheatmap [36]. Microbial profiles were displayed as bar charts (phylum level) and heatmaps (genus level, amplicon sequence variants [ASVs] >1% relative abundance).

### Statistical analyses

The prevalence of DHPV genotype V infection and its co-occurrence with EHP were calculated as percentages and analyzed with 95% confidence intervals using EpiCalc 2000 (v1.02) (Brixton Health, UK) (http://www.brixtonhealth.com/epicalc.html) and the exact binomial method [37].

Viral copy numbers from qPCR data were quantified using standard curves, and Student’s t-test was applied to assess differences in viral load between surviving and dead shrimp.

Survival data from the immersion bioassay were analyzed using Kaplan–Meier survival analysis in IBM Statistical Package for the Social Sciences Statistics v29.0.1 (IBM Corp., Armonk, NY, USA).

For microbiome analyses, alpha diversity indices were compared using the Kruskal–Wallis test, and beta diversity indices were analyzed using analysis of similarities (ANOSIM) with multiple comparisons in QIIME2.

## RESULTS

### Prevalence of DHPV in cultured *P. vannamei* and co-infection with EHP

DHPV infection was detected in 69 of 127 ponds, corresponding to a prevalence of 54.33% (95% confidence interval [CI]: 45.67–62.74). Co-infection of DHPV and EHP was recorded in 51 of 127 ponds (40.16%, 95% CI: 32.04–48.85) at grow-out farms.

EHP-infected ponds were categorized into three groups based on infection severity:

- EHP-not detected
- Light infection (EHP-positive without clinical signs)
- Heavy infection (EHP-positive with pale hepatopancreas and growth retardation).

The prevalence of DHPV infection did not differ significantly among these categories. Single DHPV infection was found in 18 of 28 ponds (64.27%, 95% CI: 45.83–79.29). The prevalence of DHPV co-infection in shrimp with light and heavy EHP infections was 46.55% (95% CI: 34.33–59.2) and 58.54% (95% CI: 43.37–72.24), respectively.

Table 1 summarizes the overall prevalence of DHPV and EHP co-infection across sampled ponds. In addition, DHPV was detected in one of 25 tanks from post-larval (PL) hatchery samples. Detailed microscopy and PCR results for all tested samples are provided in Table S2.

**Table 1:** Pond-level prevalence (%) of co-infection with DHPV and EHP in grow-out cultured shrimp in eastern Thailand (n = 127).

EHP infection	DHPV infection	

	Positive	Negative
Not detected (n = 28)		
No. of ponds (% prevalence)	18 (64.27)	10 (35.71)
95% CI	45.83–79.29	20.71–54.17
Light (n = 58)		
No. of ponds (% prevalence)	27 (46.55)	31 (53.45)
95% CI	34.33–59.2	40.8–65.67
Heavy (n = 41)		
No. of ponds (% prevalence)	24 (58.54)	17 (41.46)
95% CI	43.37–72.24	27.76–56.63
Total (n = 127)		
No. of ponds (% prevalence)	69 (54.33)	58 (45.67)
95% CI	45.67–62.74	37.26–54.33

*Light = EHP positive without clinical sign, Heavy = EHP positive with pale hepatopancreas, CI = Confidence interval, EHP = *Enterocytozoon hepatopenaei,* DHPV = Decapod hepanhamaparvovirus.

### Phylogenetic characterization of DHPV isolates

All DHPV-positive samples were classified as genotype V based on sequence analysis using the Basic Local Alignment Search Tool. Phylogenetic comparison of the representative isolates, constructed from the *NS2* and *NS1* gene regions, showed 97.99%–98.82% nucleotide similarity to DHPV strains reported from South Korea (*P. vannamei*; Accession Number ON015650.1, ON872187.1, OQ857567.1).

**Figure 1:**
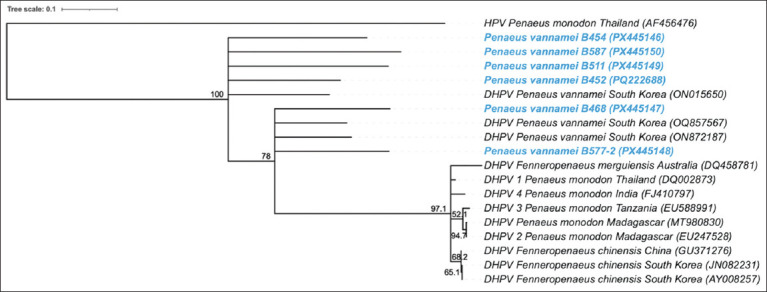
Phylogenetic analyses of six samples (B577-2, B468, B452, B511, B454, and B587) were conducted using the *NS1* and *NS2* genes, employing the neighbor-joining method and incorporating other DHPV sequences, with HPV from the National Center for Biotechnology Information database used as outgroups. Bootstrap analysis was performed using 1,000 replications. The scale bar represents 0.1 nucleotide substitutions per site. The blue color represents the DHPV samples used in this study. *NS1* = Non-structural protein 2, DHPV = Decapod hepanhamaparvovirus, HPV = Hepanhamaparvovirus.

Six isolates obtained in this study clustered within the same clade as South Korean *P. vannamei* DHPV genotype V (Figure 1). The Thai isolates also showed high similarity to DHPV detected in *P. monodon* from Madagascar (87.13%) and Tanzania (86.75%), as well as to strains from *Fenneropenaeus chinensis* in South Korea (87.79%) and China (87.42%), and *Fenneropenaeus merguiensis* in Australia (87.00%). These findings confirm that the DHPV circulating in Thailand belongs to the newly emerging genotype V lineage.

### Pathogenicity of DHPV genotype V in *P. vannamei*

**Figure 2:**
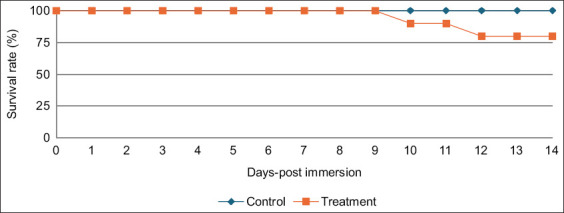
Survival of Pacific white shrimp *Penaeus vannamei* (0.4 ± 0.3 g of body weight) following immersion challenge with decapod hepanhamaparvovirus genotype V over 14 days (40 shrimp per group). The shrimp in the treatment group were exposed to 10^5^ copies/μL virus suspension in 1 L of water for 24 h. Kaplan–Meier survival analysis was performed using Statistical Package for the Social Sciences software version 29.0.1.

The experimental immersion challenge revealed a post-exposure mortality rate of 20% in the treatment group between days 10 and 12, which was not significantly different from that of the control group (Figure 2). Dead shrimp exhibited mild hepatopancreatic pallor but no other distinctive clinical signs.

All shrimp in the treatment group tested positive for DHPV by PCR at the end of the experiment. Quantitative PCR analysis indicated that moribund shrimp had significantly higher viral loads (~10³ copies/μL; Ct values 32.5–33.9) than survivors (10¹–10² copies/μL; Ct values 36.6–38.8) (p < 0.05).

**Figure 3:**
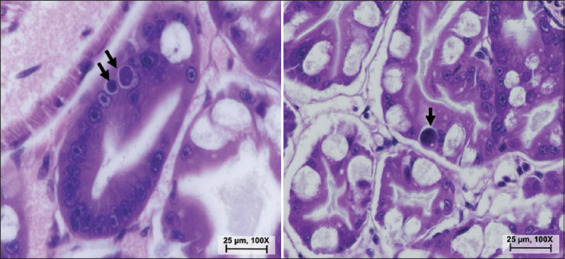
Histopathological changes revealed intranuclear inclusion bodies (arrow) in the hepatopancreatic tubular cells of decapod hepanhamaparvovirus-exposed moribund and survivors shrimp.

Histopathological examination revealed intranuclear inclusion bodies within hepatopancreatic tubular epithelial cells of both moribund and surviving challenged shrimp (Figure 3), confirming successful viral replication and low-grade pathogenicity under laboratory conditions.

### Hepatopancreatic microbiome analysis

High-throughput sequencing of *16S rRNA* amplicons produced 244,520 high-quality reads from six samples (range: 32,530–46,308 reads per sample), yielding 50 ASVs across all samples. The alpha rarefaction curve indicated that species richness reached saturation at approximately 5,000 reads (Supplementary Figure S1). Sequencing data were normalized to 30,000 reads per sample before downstream analysis.

#### Microbial diversity

**Figure 4:**
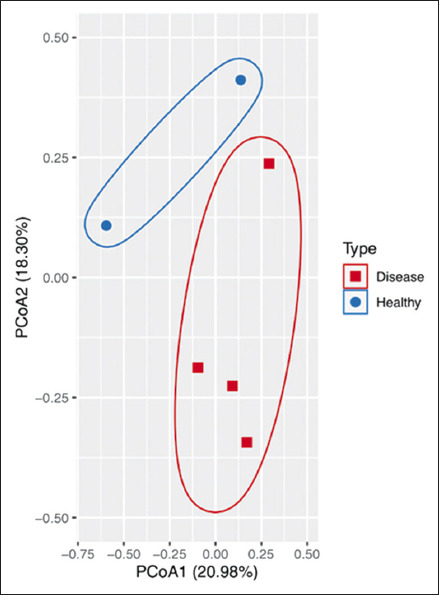
Principal coordinate analysis based on Jaccard dissimilarity displays a separation between the healthy and diseased groups. Each dot represents a sample, with red squares representing the diseased group and blue circles representing the healthy group.

The alpha diversity index (Simpson index) did not differ significantly between healthy (0.67 ± 0.41) and DHPV-infected shrimp (0.93 ± 0.05) (p > 0.05). However, beta diversity analysis using ANOSIM revealed significant community differences between the two groups (p < 0.05). PCoA based on Jaccard dissimilarity demonstrated distinct clustering of healthy and diseased hepatopancreatic microbiomes, with PCoA1 and PCoA2 explaining 20.98% and 18.30% of total variation, respectively (Figure 4).

#### Taxonomic composition

**Figure 5:**
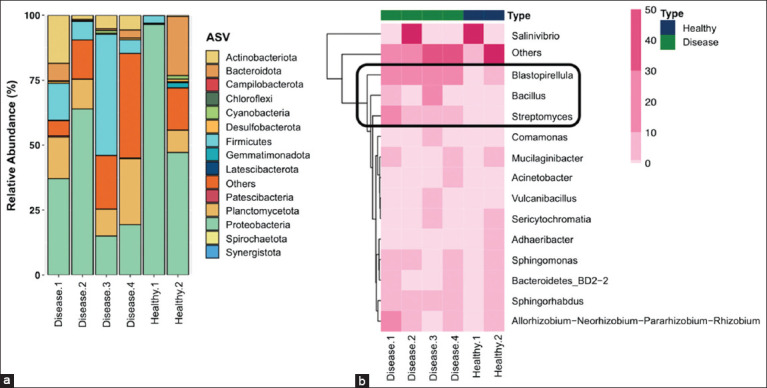
(a) Hepatopancreatic microbial profiles at the phylum level. (b) Heatmap of genus-level profiles with relative abundances >1% per sample. The key genera with biological relevance are highlighted with a black square in the heatmap figure.

Proteobacteria represented the dominant bacterial phylum in both groups. Notably, the relative abundances of Firmicutes, Planctomycetota, and Actinobacteriota were higher in DHPV-infected shrimp compared with healthy individuals (Figure 5a). At the genus level, *Bacillus*, *Blastopirellula*, and *Streptomyces* spp. were more abundant in diseased shrimp (Figure 5b). These results indicate that DHPV infection is associated with a distinct hepatopancreatic microbial profile, suggesting a potential pathobiome shift that may influence shrimp health and disease outcomes.

## DISCUSSION

### Emergence and global context of DHPV genotype V

In recent years, the impact of DHPV on shrimp health and aquaculture productivity has received growing attention. Novel genotypes of DHPV have been reported in *P. vannamei* grow-out ponds from South Korea [12, 38], Latin America [39], and India [11]. The virus has also been identified in clinically healthy post-larval *P. monodon* from Madagascar [40]. In Thailand, DHPV-like lesions have been observed in the giant river prawn *Macrobrachium rosenbergii* [14], and dual infections involving Macrobrachium hepanhamaparvovirus and DHPV have been documented [41]. However, before this study, no evidence of DHPV infection in *P. vannamei* had been reported in Thailand.

This study provides the first confirmation of DHPV genotype V in Thai *P. vannamei*, an economically critical aquaculture species. Furthermore, by integrating virological and microbiome analyses, this research offers novel insights into host–pathogen–microbiota interactions that may underlie shrimp health and performance.

### Epidemiology and co-infections

DHPV genotype V infection was detected in *P. vannamei* cultured in the eastern region of Thailand, confirming its circulation in commercial shrimp farms. DHPV was also detected in apparently healthy PL from a southern hatchery, suggesting potential contamination during spawning or larval rearing through contact with fomites or infected broodstock. Although vertical transmission of historical hepatopancreatic parvovirus (HPV) has not been demonstrated [42], these findings indicate that early-life exposure to DHPV cannot be ruled out.

The detection of DHPV in both healthy and diseased shrimp underscores the possibility of covert infections and asymptomatic carriers contributing to virus persistence within intensive farming systems. A high rate of DHPV–EHP co-infection was observed in grow-out shrimp. As EHP infection is known to cause chronic growth retardation and physiological stress, it may predispose shrimp to secondary viral infections, including DHPV [43].

Similar patterns of DHPV–EHP co-infections have been reported globally, with incidences reaching 71.4% for EHP and 49.4% for WSSV in South Korea, and DHPV–EHP co-infections in Taiwan farms [12, 18, 44]. The novel DHPV genotype V possesses unique deletions and insertions in the VP gene, which may influence its virulence and interactions with co-infecting pathogens [44].

Given that both EHP and DHPV target the hepatopancreas, a key organ for digestion, nutrient absorption, and metabolism, their co-infection could have synergistic pathological impacts. This warrants further experimental co-challenge studies to clarify their combined effects on shrimp physiology and productivity. In addition, cross-species transmission risks between *M. rosenbergii* and *P. vannamei*, which share similar rearing environments, must be evaluated as part of One Health and biosecurity frameworks to prevent pathogen dissemination through hatchery and trade networks.

### Phylogenetic characterization and genetic relationships

Comparative sequence analysis revealed that DHPV isolates from this study shared 97.99%–98.82% similarity with DHPV strains from *P. vannamei* in South Korea and Latin America, confirming their classification as genotype V [38, 39]. The gene regions analyzed included non-structural proteins *NS1* and *NS2*, with the highest nucleotide diversity detected in the *VP* gene, consistent with previous findings [40, 45].

The specific *NS2*–*NS1* junction fragment targeted in this study corresponds to a highly conserved region under purifying selection, ensuring accurate detection and differentiation from historical HPV strains [22]. Phylogenetic clustering placed Thai isolates in a distinct lineage separate from traditional HPV clades, suggesting regional or transboundary viral spread likely facilitated by international broodstock and larval trade.

Geographical and ecological variables, host species composition, and farming practices (e.g., mixed culture of *P. monodon* and *P. vannamei*) may contribute to viral diversification and host adaptation [46]. Comprehensive whole-genome sequencing of DHPV genotype V from diverse hosts and regions will enhance global epidemiological mapping and evolutionary understanding of this emerging pathogen in shrimp aquaculture.

### Pathogenicity and histopathological features

Experimental immersion challenge confirmed DHPV infection and active replication in *P. vannamei*, as evidenced by qPCR quantification and characteristic histopathological lesions. Although overall mortality was low (20%) and not significantly different from that of controls, infected shrimp exhibited mild hepatopancreatic pallor and intranuclear basophilic inclusion bodies within the E- and F-cells of hepatopancreatic tubules, particularly in distal regions. These inclusions serve as reliable histological markers of DHPV infection [47, 48].

The low virulence observed aligns with reports from South Korea, where DHPV genotype V produced similar lesions without causing severe mortality [12]. In contrast, *P. monodon* PL from Madagascar infected with DHPV did not display any histopathological changes [40], suggesting possible host-specific responses or viral attenuation.

Despite its low pathogenicity, DHPV was frequently detected in growth-retarded shrimp, both with and without EHP co-infection. This supports the hypothesis that DHPV may cause subclinical infections contributing to chronic production losses. When co-infecting with EHP or other pathogens such as infectious myonecrosis virus (IMNV), DHPV could exacerbate hepatopancreatic dysfunction and retardation, emphasizing the need for long-term surveillance and risk assessment in breeding and grow-out systems.

### Hepatopancreatic microbiome alterations and pathobiome perspective

Amplicon-based *16S rRNA* sequencing of hepatopancreatic samples revealed notable alterations in microbial community structure between DHPV-infected and healthy shrimp. The study targeted the V3–V4 hypervariable regions, known for their superior taxonomic resolution in shrimp microbiome studies [49].

Although alpha diversity indices showed no significant difference between groups, beta diversity analyses (PCoA and ANOSIM) demonstrated clear community separation, suggesting that DHPV infection influenced microbiome composition more than richness. Similar findings were observed in *Procambarus clarkii* exhibiting disease symptoms [50], as well as in several viral and bacterial infections in *P. vannamei* and *P. monodon* [51–55].

Proteobacteria was the dominant phylum in both healthy and diseased shrimp, consistent with reports from wild and farmed *P. vannamei* [53]. However, infected shrimp displayed higher relative abundances of Firmicutes, Planctomycetota, and Actinobacteriota, a pattern commonly linked to disease-associated dysbiosis [56–58]. At the genus level, *Bacillus*, *Blastopirellula*, and *Streptomyces* spp. were enriched in DHPV-infected shrimp. Notably, *Bacillus* and *Streptomyces* spp. are recognized as beneficial probiotics with tolerance to environmental stressors and antimicrobial properties [59, 60]. Their increased abundance might represent a compensatory or adaptive microbial response to viral stress within the hepatopancreas.

Collectively, these findings suggest that DHPV infection contributes to pathobiome shifts, altering the hepatopancreatic microbial equilibrium. This integrative approach combining virological and microbiome analyses provides a holistic understanding of disease ecology, aligning with the One Health paradigm and supporting aquaculture sustainability.

## CONCLUSION

This study provides the first molecular evidence of DHPV genotype V infection in *P. vannamei* cultured in Thailand, confirming its widespread distribution in grow-out ponds and occasional detection in hatchery PL. The overall DHPV prevalence was 54.33%, with frequent co-infection with EHP (40.16%). Phylogenetic analysis revealed 97.99%–98.82% similarity with DHPV strains from South Korea and Latin America, suggesting transboundary dissemination through international broodstock and larval movement. Experimental immersion trials confirmed viral replication and the presence of intranuclear inclusion bodies in hepatopancreatic cells, indicating active infection with low virulence. Although mortality was limited (20%), the findings highlight potential subclinical effects on shrimp health and growth. Microbiome profiling revealed distinct compositional shifts in DHPV-infected shrimp, including increased abundances of Firmicutes, Planctomycetota, and Actinobacteriota, indicating a virus-associated pathobiome alteration.

The practical implications of this work are significant for disease surveillance and management. Routine molecular screening of broodstock, PL, and farmed shrimp is strongly recommended to detect covert infections. Integration of virological and microbiome analyses can improve early detection and biosecurity strategies. Hatchery-level interventions, such as disinfection, quarantine, and improved water-management practices, can minimize horizontal transmission risks. Recognition of DHPV–EHP co-infections as a potential cause of chronic growth retardation may guide nutrition and management strategies to mitigate economic losses.

The main strengths of this study lie in its integrative design, which combines epidemiological, molecular, pathological, and microbiome approaches, providing a holistic perspective on DHPV ecology. However, the limited sample size for microbiome sequencing and the absence of controlled co-infection challenge trials constitute key limitations. Future research should focus on DHPV–EHP synergistic mechanisms, host immune modulation, and whole-genome sequencing to elucidate viral evolution and spread.

Overall, this study establishes a scientific foundation for managing DHPV in Thai shrimp aquaculture. The findings contribute to the United Nations SDGs 14 (Life Below Water) and 2 (Zero Hunger) by promoting sustainable, biosecure, and resilient shrimp-farming systems.

## DATA AVAILABILITY

The supplementary data can be made available from the corresponding author upon request.

## AUTHORS’ CONTRIBUTIONS

OC and PT: Conceptualization, methodology, investigation, data curation, formal analysis, and drafted and edited the manuscript. PS: Methodology, investigation, and data curation. HJJ and BK: Methodology, investigation, data curation, and formal analysis. JEH: Visualization, supervision, and writing – review and editing of the manuscript. PP: Conceptualization, investigation, data curation, formal analysis, visualization, supervision, and writing – review and editing of the manuscript. All authors have read and approved the final version of the manuscript.
